# Draft Genome Sequences of 14 *Escherichia coli* Phages Isolated from Cattle Slurry

**DOI:** 10.1128/genomeA.01364-15

**Published:** 2015-12-31

**Authors:** R. Smith, M. O’Hara, J. L. Hobman, A. D. Millard

**Affiliations:** a School of Life Sciences, University of Warwick, Coventry, United Kingdom; b School of Biosciences, University of Nottingham, Sutton Bonington Campus, Sutton Bonington, Leicestershire, United Kingdom; c Microbiology and Infection Unit, Warwick Medical School, University of Warwick, Coventry, United Kingdom

## Abstract

The diversity of bacteriophages in slurry from dairy cows remains largely unknown. Here, we report the draft genome sequences of 14 bacteriophages isolated from dairy cow slurry using *Escherichia coli* K-12 MG1655 as a host.

## GENOME ANNOUNCEMENT

There are approximately 1.8 million head of dairy cattle in the United Kingdom, producing a total of ~14 billion liters of milk per annum. Millions of tonnes of manure are produced by the UK dairy herd. The solid manure and liquid cow slurry produced by these animals is widely used as fertilizer and has the potential to allow the transmission of bacteria into the environment. There is concern that cattle slurry will allow the transmission of pathogenic bacteria and antibiotic resistance genes into soils (1), which ultimately may enter the human food chain. While the bacterial fraction of cattle slurry has been studied (2), the viral fraction has not. Here, we examined the diversity of bacteriophages capable of infecting *Escherichia coli*. A single slurry sample from a dairy farm slurry tank in the East Midlands of the United Kingdom was collected. The titer of phage capable of infecting *Escherichia coli* MG1655, was 4.43 × 10^2^ (±1.2 × 10^2^) PFU/ml. A total of 30 bacteriophages were isolated, and 14 independent bacteriophage isolates were chosen at random for genome sequencing. Bacteriophage isolates were purified by a double overlay plaque assay (3). DNA was extracted from 1 mL of crude lysates using a modified phenol:chloroform extraction method (4). One nanogram of input DNA was used to prepare a genomic library for sequencing, using the Illumina Nextera XT DNA sample kit per the manufacturer’s protocol (Illumina, USA). Sequencing was performed on an Illumina MiSeq instrument using the paired-end 2 × 250-bp protocol (version 2). Reads were trimmed with Sickle (5); genomes were assembled using SPAdes 3-1 with the “only-assembler” option (6). The sequence was further checked for errors by mapping reads against the resulting assembly with BWA-MEM using default settings, to correct any miscalled bases (7).

The remapping of sequences allowed the complete circularly permuted genomes to be identified (slur02, slur03, slur04, slur07, slur08, slur11, slur13, and slur14). The exact termini of the remaining phages have not been experimentally determined. Assembled genomes were annotated with Prokka version 1.11 (8) using a custom database constructed from all viral genomes available from EBI at the time. Bacteriophage genomes ranged in size from 43.9 kb (slur05) to 167.467 kb (slur08), and the G+C content ranged from 35.4% (slur14) to 54.5% (slur05). The most common type of bacteriophage isolated belonged to the genus *T4likevirus*, with eight bacteriophage isolates (slur02, slur03, slur04, slur07, slur08, slur11, slur13, and slur14) within this genus. In comparison to the archetypal phage T4, these isolates contained far fewer endonuclease-encoding genes (e.g., *segD*, *segC*, *mobA*, and *mobB*). Homologues of the *b-gt* gene that encodes for β-glucosyltransferase, which glucosylates phage DNA and provides protection from host restriction enzymes, were also absent. Two HK578-like viruses (slur05 and slur06), along with two phages closely related to bacteriophage rv5 (slur16 and slur12), a T5-like phage (slur09) and a single phage (slur01) that had a high similarity to the previously novel enterotoxigenic *E. coli* phage Seurat (9), were also isolated. This collection of phages expands our knowledge of phages associated with cattle slurry.

### Nucleotide sequence accession numbers.

This whole-genome shotgun project has been deposited at DDBJ/EMBL/GenBank under the accession numbers LN881725 (slur01), LN881726 (slur02), LN881728 (slur03), LN881729 (slur04), LN881730 (slur05), LN881731 (slur06), LN881732 (slur07), LN881733 (slur08), LN887948 (slur09), LN881734 (slur11), LN881735 (slur12)**,** LN881737 (slur 13), LN881736 (slur14), and LN881727 (slur16).
